# Association of lipid profile with bone mineral density in postmenopausal women in Yazd province

**Published:** 2016-09

**Authors:** Akram Ghadiri-Anari, Zahra Mortezaii-Shoroki, Mozhgan Modarresi, Ali Dehghan

**Affiliations:** 1 *Diabetes Research Center, Shahid Sadoughi University of Medical Sciences, Yazd, Iran.*; 2 *School of Medicine, Shahid Sadoughi University of Medical Sciences, Yazd, Iran.*; 3 *Department of Community Medicine, School of Medicine, Shahid Sadoughi University of Medical Sciences, Yazd, Iran.*; 4 *Department of Internal Medicine, Shahid Sadoughi University of Medical Sciences, Yazd, Iran.*

**Keywords:** *Cholesterol*, *Osteoporosis*, *Postmenopausal women*, *Lipids*, *Bone mineral density*

## Abstract

**Background:**

Low bone mass is a major health problem in postmenopausal women. There is no general agreement regarding relationship between serum level of lipids and bone mineral density.

**Objective:**

This study was carried out to investigate the association between lipid profile and bone mineral density (BMD) in postmenopausal women in Yazd, Iran.

**Materials and Methods:**

This cross-sectional study was performed on 170 women aged between 50 and 70 years old with menopause for at least one year from Yazd, Iran, between March 2013 to September 2013. Association of lipid profile and BMD were measured in all study participants.

**Results:**

Among our participants 73 cases had lumbar osteoporosis, 17 cases had femoral osteoporosis and 80 cases did n’t have osteoporosis. After controlling for body mass index, there were no correlations between serum level of lipids and bone mineral density of femur and lumbar bones.

**Conclusion:**

No significant association between serum level of lipids and BMD of femur and lumbar was found in postmenopausal women.

## Introduction

Osteoporosis as a metabolic bone disorder is accompanied with low mass and weakness of the bones. Dyslipidemia has been associated with low bone mineral density in some studies, but other studies such as Samelson *et al*, Solomon *et al*, and Wu *et al* found no relationship between total serum cholesterol levels and bone mineral density (BMD) (1-6). Some studies have shown that subjects with osteoporosis have a higher risk of cardiovascular events resulting from atherosclerosis than those with osteopenia (7). Low-density lipoprotein (LDL) receptor- related protein 5 (LRP5) deficient mice were shown to have both hypercholesterolemia and bone mass reduction (8). 

Family members with mutation of LRP6 in humans were shown to have both early-onset cardiovascular diseases and severe osteoporosis complicated by high serum low-density lipoprotein cholesterol (LDL-C) levels, hypertension, and impaired glucose tolerance, suggesting that these components of the metabolic syndrome could cause bone fragility, as well as atherosclerosis (9). Osteoporosis is a major health problem in postmenopausal women. Osteoporotic women are at risk of cardiovascular disease and stroke having higher atherogenic lipid levels than women without this problem (10). 

Due to the controversy on the previous studies and lack of data in Iranian postmenopausal women, we investigated the possible relationships between serum lipid profile and BMD in this group in Yazd, Iran. Also the level of lipids between osteoporotic and non-osteoporotic women was compared.

## Materials and methods

This cross-sectional study was performed on 170 women who came to bone mineral densitometry clinic from Yazd, Iran, between March 2013 to September 2013. Women were enrolled between 50 and 70 years old, as well as being menopause for at least one year. All participants signed a consent form before participating in this study and their demographic information was collected by a questionnaire. Also study protocol was approved by the ethics committee of Shahid Sadoughi University of Medical Science, Yazd, Iran.

Patients were excluded if they were taking drugs such as statins, vitamin D, bisphosphonates, glucocorticoids and high dose of levothyroxine*.* Also patients with renal failure, chronic infections such as tuberculosis, diabetes mellitus, malignancy, osteomalacia, and thyrotoxicosis were excluded from the study. History of smoking, alcohol abuse, hypertension, and low trauma fracture was recorded. Physical examination conducted of each participant. Height was measured to the nearest 0.1 cm on a portable stand meter, and weight was measured to the nearest 0.1 kg with the subject standing motionless in the center of the scale.

Weight and height were measured without the subjects’ wearing shoes. Body mass index (BMI) was calculated as weight/height^2^. Blood samples were collected after 12 hrs fasting for triglyceride, total cholesterol, LDL, and HDL levels. Serum total cholesterol, triglyceride and HDL-C levels were measured by enzymatic methods (Pars-Azmoon, Karaj, Iran). LDL-C calculated using the Friedewald formula: LDL-cholesterol=Total cholesterol- (TG/5 + HDL-C) if triglyceride concentration is below 400 mg/dl (11). Dual-energy X-ray absorptiometry was used to measure bone mineral density (BMD, in g/cm^2^) in proximal femur and lumbar spine using Hologic Inc. ASY00409 X-Ray Controller for Hologic Discovery Bone Densitometry.

The osteoporosis was defined as a total hip or lumbar spine BMD of ≥2.5 SDs below the average peak BMD of young age, healthy women (T-Score ≤-2.5). The results of densitometry including BMD, T-score, Z-score were also recorded. Lipid profile of osteoporotic women was compared with those who had normal bone density.

**Statistical analysis**

Mann-Whitney U-test and T-Test and Pearson's correlation coefficients were used for analysis of data. The normalized data were analyzed with T-Test. Others analyzed by Mann-Whitney U-test. P≤0.05 was considered significant. All statistical analyses were performed using SPSS software version 18.

## Results

In this study, 170 female who had been postmenopausal for at least one year with mean age of 61.80±7.44 yrs old (50-70 yrs old) entered. Basic characteristics of participants are given (Table I). Among our participants, 73 cases had lumbar osteoporosis, 17 cases had femoral osteoporosis and 80 cases had no osteoporosis. One female had osteoporotic fracture. All participants had no history of smoking or alcohol abuse. Mean triglyceride was 168±90mg/dl and mean of total cholesterol was 214±38 mg/dl. Means of HDL-C and LDL-C were 54±12 mg/dl and 132±32 mg/dl, respectively. 

Mean of lumbar and femoral bone mineral density (BMD) in our study was 0.79±0.13 g/cm^2^ and 0.69±0.13 g/cm^2^ respectively. We found unadjusted negative correlation between serum level of total cholesterol with femur BMD (p=0.037) but when linear regression models adjusted for weight and BMI were applied, to explore the magnitude of relation between serum lipid profile and bone mineral density, we found no correlation between serum level of total cholesterol and femoral BMD. 

These data are summarized in tables II and III. Mean of lipid levels did not show statistically significant difference between osteoporotic and non-osteoporotic women. These data are shown in table IV. 

**Table I:** Descriptive Characteristics of the Study Population

	**Study population**
Age (yr)	61.80 ± 7.44
Weight (kg)	69 ± 9.9
Height (cm)	155 ± 5.8
HDL-C (mg/dl)	54 ± 12.8
LDL-C (mg/dl)	132 ± 32.6
TG (mg/dl)	168 ± 90.9
Total cholesterol l(mg/dl)	214 ± 38.3
Lumbar BMD (g/cm^2^)	0.79 ± 0.13
Lumbar T score	-2.2 ± 1.2
Lumbar Z score	-0.86 ± 1.1
Femoral BMD (g/cm^2^)	0.69 ± 0.13
Femoral T score	-1.4 ± 1.01
Femoral Z score	-0.16 ± 0.93

Data are presented as Mean±SD.

HDL-C: High Density Lipoprotein- Cholesterol

LDL-C: Low Density Lipoprotein- Cholesterol

TG: Triglycerides

Lumbar BMD: Lumbar Bone Mineral Density

Femoral BMD: Femoral Bone Mineral Density

**Table II:** Association Between lipid profile and bone mineral density (un-adjusted

**Lipid profile**	**p-value**	
	**Lumbar BMD**	**Femoral BMD**
HDL-C	0.22	0.54
LDL-C	0.58	0.051
TG	0.80	0.627
Total Cholesterol	0.12	0.037

Data are presented as Pearson Correlation.

HDL-C: High Density Lipoprotein- Cholesterol

LDL-C: Low Density Lipoprotein- Cholesterol

TG: Triglycerides

**Table III:** Regression coefficients (β) for serum lipids (mg/dl) to lumbar BMD (g/cm^2^) and Femoral BMD (g/cm^2^

**Variable**	**Lumbar BMD**	**P value**	**Femoral BMD**	**p-value**
HDL-C	-0.046 ± 0.001	0.54	0.005 ± 0.001	0.95
LDL-C	0.173 ± 0.000	0.16	-0.033 ± 0.001	0.78
TG	0.035 ± 0.000	0.62	0.056 ± 0.000	0.43
Total Cholesterol	-0.238 ± 0.000	0.06	-0.130 ± 0.000	0.31

All Data are presented as β±SE. The regression models were adjusted for weight and body mass index.

HDL-C: High Density Lipoprotein- Cholesterol

LDL-C: Low Density Lipoprotein- Cholesterol

TG: Triglycerides

**Table IV:** Comparison of lipid profile between osteoporotic and non-osteoporotic women

	**Femoral**			**Lumbar**		
	**Osteoporotic**	**Non-osteoporotic**	**p-value**	**Osteoporotic**	**Non-osteoporotic**	**p-value**
LDL-C	134 ± 35	132 ± 32	0.746	135 ± 37.4	130 ± 28.5	0.325
HDL-C	56.5 ± 13	54.3 ± 13	0.426	56 ± 12.1	53.4 ± 13.3	0.067
TG	151 ± 75	170 ± 92.6	0.354	160 ± 103	175 ± 80.4	0.073
Total Chol	220 ± 34.3	213 ± 38.8	0.474	219 ± 40	210 ± 36.7	0.446

Data are presented as mean±SD.

Mann Whitney U test.

HDL-C: High Density Lipoprotein- Cholesterol

LDL-C: Low Density Lipoprotein- Cholesterol

TG: Triglycerides

## Discussion

Lipid disorders have been associated with low bone mineral density in some studies (1-3). The mechanism of this relation may be directly related with the cholesterol biosynthetic pathway, which determines cholesterol levels and contributes to the activity of the osteoclast (12). Beneficial effect of lipid reducing drugs such as statins on bone mineral density has been seen in most of previous studies (13-15). These findings proposed the probable association between serum lipid profile and BMD especially among patients with increased risk of osteoporosis rather than healthy persons (16-17). 

Parhami et al. declared that a baseline level of cholesterol synthesis is necessary for the osteoblastic differentiation of marrow stromal cells (18). Low-density lipoprotein (LDL) receptor-related protein 5 (LRP5) deficient mice were shown to have both hypercholesterolemia and bone mass reduction (8). Mutation of LRP6 in humans were shown to cause both early-onset cardiovascular diseases and severe osteoporosis complicated by high serum low-density lipoprotein cholesterol (LDL-C) levels, suggesting that this mutation could cause bone fragility, as well as atherosclerosis (9).

We found unadjusted negative correlation between serum levels of total cholesterol with femoral BMD. When linear regression models were adjusted for weight and BMI, we found no correlation between serum level of lipids and BMD. Also lipid levels did not show significant difference between osteoporotic and non-osteoporotic women. 

Association of lipid profile and BMD has been evaluated in several studies, but the reports are controversial. Some studies have supported the negative correlation (1-3). For example, Orozco and coworkers evaluated fifty-two overweight early postmenopausal women and found that postmenopausal women with atherogenic lipid profile, defined as high cholesterol or LDL-c above 160 mg/dl or high lipoprotein (a) have lower lumbar and femoral BMD and have an increased risk of osteopenia than those with normal lipid profile (1). Also, there are some studies that have found positive correlation between serum lipids and BMD (19-20). For example, Brownbill *et al* evaluated 136 Caucasian, healthy, postmenopausal women and found that higher levels of serum triglycerides and cholesterol are positively associated with BMD of various skeletal sites (17). 

Also, some studies reported no correlation between serum lipids and BMD (4-6). For instance, Solomon investigated a large number (13592 participants) and did not find any association between serum lipid profile and BMD (4). This study is noticeable by considering its sample size. Also Samelson in a prospective study found that women and men with increased total cholesterol levels throughout young to middle adult years had similar BMD with increasing age as those who had lower cholesterol levels (5). Framingham Osteoporosis Study conducted over a long period of follow-up with a large number of repeated measures of cholesterol and potential confounders (5). In addition, this study measured bone mineral density at several bone sites and in both women and men selected from a population based sample (5). 

Taken together with the large sample size and large number of repeated measures, the lack of association between total cholesterol and BMD is not likely explained by inadequate study power or measurement error. The result of our study is similar to the last studies that have large sample size (4-6). Age, gender, menopausal status and BMI are making change in bone mineral density and lipids. Thus, lack of consensus findings among these studies may be affected by different ethnic of the participants in each study, background diseases or medical conditions such as vitamin-D insufficiency or alcohol consumption that are known to be associated with osteoporosis.

In our study we found unadjusted negative correlation between serum levels of total cholesterol and femoral BMD but these relationships were greatly attenuated when adjusted for BMI and weight. BMI and weight are important factors that change both bone mineral density and lipids. Relationship between bone mineral density and lipids is manipulated by many various factors such as age, lifestyle, physical activity, consumption of dairy products and amount of fat mass that all of them should be considered. Also cross-sectional nature of this study and the age of the participants that is from early postmenopausal period may affect these results. Future studies with older women that probability of osteoporosis and atherosclerosis is high, may be necessary. 

**Limitations**

First the sample size was relatively small and not large enough to cause adequate study power to our results. Second, repeat measurement of lipids maybe necessary for decreasing confounding factors such as diets on lipid profile. Also, next studies with both male and female sex are recommended and the level of physical activity of participant must be determined.

## Conclusion

There is no association between serum level of lipids and bone mineral density of femur and lumbar in postmenopausal women in this study.

## Note

***This article extracted from M.D. thesis. (Zahra Mortezaii-Shoroki)***
